# Genomic Imprinting

**DOI:** 10.3889/oamjms.2016.028

**Published:** 2016-02-04

**Authors:** Emirjeta Bajrami, Mirko Spiroski

**Affiliations:** 1 *University Clinical Centre, Neonatology Clinic, Prishtina, Kosovo*; 2 *Institute of Immunobiology and Human Genetics, Faculty of Medicine, Ss Cyril and Methodius University of Skopje, Skopje, Republic of Macedonia*

**Keywords:** genomic imprinting, epigenetic inheritance, gene, DNA methylation

## Abstract

**BACKGROUND:**

Genomic imprinting is the inheritance out of Mendelian borders. Many of inherited diseases and human development violates Mendelian law of inheritance, this way of inheriting is studied by epigenetics.

**AIM:**

The aim of this review is to analyze current opinions and options regarding to this way of inheriting.

**RESULTS:**

Epigenetics shows that gene expression undergoes changes more complex than modifications in the DNA sequence; it includes the environmental influence on the gametes before conception. Humans inherit two alleles from mother and father, both are functional for the majority of the genes, but sometimes one is turned off or “stamped” and doesn’t show in offspring, that gene is imprinted. Imprinting means that that gene is silenced, and gene from other parent is expressed. The mechanisms for imprinting are still incompletely defined, but they involve epigenetic modifications that are erased and then reset during the creation of eggs and sperm. Genomic imprinting is a process of silencing genes through DNA methylation. The repressed allele is methylated, while the active allele is unmethylated. The most well-known conditions include Prader-Willi syndrome, and Angelman syndrome. Both of these syndromes can be caused by imprinting or other errors involving genes on the long arm of chromosome 15.

**CONCLUSIONS:**

Genomic imprinting and other epigenetic mechanisms such as environment is shown that plays role in offspring neurodevelopment and autism spectrum disorder.

## Introduction

Genomic imprinting is the inheritance out of Mendelian borders. Many of inherited diseases and human development violates Mendelian law of inheritance, this way of inheriting is studied by epigenetics. Epigenetics shows that gene expression undergoes changes more complex than modifications in the DNA sequence; it includes the environmental influence on the gametes before conception.

When epigenetic changes occur in sperm or egg cells that lead to fertilization, epigenetic changes are inherited by the offspring [1].

Genomic imprinting is a process of silencing genes through DNA methylation. The repressed allele is methylated, while the active allele is unmethylated.

Some questions still await conclusive answers, particularly those concerning why mammals alone among vertebrates use imprinted genes to regulate embryonic and neonatal growth [2].

The aim of this review is to analyze current opinions and options regarding to this way of inheriting.

## Results and Discussion

The classical definition of epigenetics refers to the mitotically and/or meiotically heritable changes in gene activity that does not involve alterations in DNA sequence [3]. Genomic imprinting occurs when two alleles at a locus are not functionally equivalent and is considered the primary epigenetic phenomenon that can lead to the manifestation of parent-of-origin effects [4]. Genomic imprinting affects both male and female offspring and is therefore a consequence of parental inheritance, not of sex [2]. Epigenetic changes can be induced by environmental factors at different times in life. Epigenetic control operates on three major levels, on DNA, histones, and nucleosomes [3]. Epigenetic mechanisms encode information above and beyond DNA sequence and play a critical role in brain development and the long-lived effects of environmental cues on the pre- and postnatal brain [5] and [6].

When epigenetic changes occur in sperm or egg cells that lead to fertilization, epigenetic changes are inherited by the offspring [1].

Genomic imprinting is a process of silencing genes through DNA methylation. The repressed allele is methylated, while the active allele is unmethylated. This stamping process, called methylation, is a chemical reaction that attaches small molecules called methyl groups to certain segments of DNA [3]. DNA methylation is a biochemical process crucial for normal development in higher organisms, and it is the most thoroughly studied epigenetic mark. Methylation entails the covalent attachment of a methyl (CH_3_) group to the C5 position of a cytosine residue, forming 5-methylcytosine (5 mC) [3]. DNA methylation is mediated by the cellular DNA methylation machinery, comprising Dnmt1, Dnmt3a, Dnmt3b and Dnmt3L. DNA methylation is a dynamic process during early embryonic development and undergoes parent and lineage dependent genome-wide changes [3] and [7].

There are now more than 25 identified imprinted genes, and estimates based on mouse models indicate that as many as 100 to 200 may exist [8]. The first endogenous imprinted gene identified was mouse insulin-like growth factor 2 (Igf2), which encodes for a critical fetal-specific growth factor [8] and [9].

Many theories have attempted to explain the evolution of genomic imprinting, but the most prominent are the kinship theory [10] and the sex-specific selection theory [11]. The kinship theory relies on asymmetries in relatedness between individuals’ maternally and paternally derived alleles [12]. The kinship theory predicts that genes increasing an offspring’s share of maternal resources, such as growth enhancers that act in development, will be expressed from the paternally derived allele and repressed on the maternally derived allele [13]. For X-linked loci, inheritance is asymmetric with respect to parental origin, and imprinting allows expression from such loci to be sexually dimorphic [10]. Under weak selection, quantitative genetic models of X-linked loci suggest that when selection is stronger against one sex, expression in the offspring of alleles derived from the other sex should be higher [10].

Although the exact molecular mechanisms involved in establishing and maintaining genomic imprints remain undetermined, much is known about the basic details [14]. Imprinted genes often occur in clusters that contain one or more imprinting control regions (ICRs). ICRs often exhibit different patterns of DNA methylation depending on whether the allele is paternally or maternally inherited [15]. The parental allele-specific epigenetic marks are heritable to the daughter cells, but must be reset in each successive generation to establish parental specific imprints. In mammals, two major genome-wide epigenetic reprogramming events take place during gametogenesis and early embryogenesis [15].

How does transcription lead to DNA methylation in oocytes? Oocyte availability is a challenge to molecular studies, but Kelsey and Feil [16] have speculated that the act of transcription results in a constellation of chromatin modifications that are conducive to interaction of DNMT3A and DNMTL, whereas other transcribed regions might be protected from methylation by CXXC-domain proteins.

Genomic imprints template their own replication, are heritable, can be identified by molecular analysis, and serve as markers of the parental origin of genomic regions. Beyond merely labeling homologous genetic alleles as descendent from father or mother, genomic imprints have the significant functional consequence of stifling gene expression from one of the parental alleles, resulting in unbalanced gene expression between homologous alleles.

### The life cycle of imprints

Genomic imprints change in characteristic ways during the life cycle of the organism [17] and [18]. Imprints are ‘established’ during the development of germ cells into sperm or eggs. After fertilization, they are ‘maintained’ as chromosomes duplicate and segregate in the developing organism. In the germ cells of the new organism, imprints are ‘erased’ at an early stage [17]. This is followed by establishment again at a later stage of germ-cell development, thus completing the imprinting cycle. In somatic cells, imprints are maintained and are modified during development [17]. The imprints that are introduced in the parental germlines, maintained in the early embryo and fully matured during differentiation, they need to be read. Reading means the conversion of methylation or chromatin imprints into differential gene expression [17] and [18]. As a result of imprinting, there is biased allelic expression that favors expression from one parental locus over the other.

The dispersed patterns of CpG dyads in the early-cleavage embryo suggest a continuous partial (and to a low extent active) loss of methylation apparently compensated for by selective de novo methylation [18] and [19]. A combination of passive and active demethylation events counteracted by de novo methylation are involved in the distinct reprogramming dynamics of DNA methylomes in the zygote, the early embryo, and PGCs [19].

### Imprinted genes code for what?

A majority of the known imprinted genes code for proteins, others code for untranslated RNA transcripts.

Another category of parental genomic imprint, to be contrasted with well characterized examples of monoallelically expressed genes, are those methylation parental imprints scattered throughout the genome which are not demonstrated to be functional or associated with specific genes [18].

Clusters of imprinted genes are often controlled by an imprinting center that is necessary for allele-specific gene expression and to reprogram parent-of-origin information between generations. An imprinted domain at 15q11–q13 is responsible for both Angelman syndrome and Prader–Willi syndrome, two clinically distinct neurodevelopmental disorders [20].

The imprinted gene cluster on 15q11–q13 contains a number of paternally and maternally expressed transcripts and is reasonably well conserved, in terms of both gene content and imprinting status, between mammals [21] and [22]. The cluster has been studied intensely as loss of expression, through genetic and epigenetic mutation, leads to two distinct neurodevelopmental disorders, namely Prader- Willi Syndrome, which results as a consequence of loss of paternal gene expression, and Angelman Syndrome, which arises as a consequence of loss of maternal gene expression [22] and [23].

Prader-Willi syndrome is characterized by abnormal feeding and appetite, and learning disability, individuals with PWS may also develop a severe affective psychotic illness which is similar to bipolar disorder. This includes loss of antisense transcripts which represses the expression of UBE3A, which encodes E6-AP (E6-associated protein) ubiquitin ligase from the paternal chromosome. As a consequence, the paternal copy of this gene, which is only normally expressed from the maternal chromosome, becomes reactivated leading to increased dosage [22].

AS is a neurodevelopmental disorder characterized by severe cognitive disability, motor dysfunction, speech impairment, hyperactivity, and frequent seizures. AS is caused by disruption of the maternally expressed and paternally imprinted UBE3A, which encodes an E3 ubiquitin ligase.

In addition to AS and PWS, the 15q11–q13 imprinting region has also been linked to a number of non-syndromic neuropsychiatric illnesses. For instance, maternal duplication of this interval is associated with the incidence of autism [24].

Several studies have reported differential expression of imprinted genes between control and IUGR placental samples [24]. In other words, some may act to reduce fetal growth, resulting in IUGR (negative effectors), while others may act to enhance fetal growth in a compensatory manner to save a pathogenically growth restricted fetus (positive effectors) [25].

Some questions still await conclusive answers, particularly those concerning why mammals alone among vertebrates use imprinted genes to regulate embryonic and neonatal growth [2]. At this stage, it is clear that genomic imprinting uses the cell’s normal epigenetic machinery to regulate parental-specific expression, and that everything is set in motion by restricting this machinery in the gamete to just one parental allele [2]. An improved understanding of genomic imprinting will undoubtedly continue to provide an important model to discover how the mammalian genome uses epigenetic mechanisms to regulate gene expression [2].

In conclusoon, genomic imprinting is important process of inheritance that plays important role in future genetic studies. It is a complex process that is based on DNA metylation in alleles of chromosomes. Numerous external cues influence DNA methylation, which may determine disease onset or progression. Genomic imprinting is a fairly rare phenomenon in humans, most genes are not imprinted, and most of studies are done in mice or plants, so we have a lot to do in this field. Although we do not yet know the precise mechanisms underlying epigenetic gene regulation in the pathogenesis of several diseases, there are finding that the progression of such diseases can be altered by modulating epigenetic programs.
